# ‘Why didn’t they see my scars?’ Critical thematic analysis of simulated participants’ perceived tensions surrounding objective structured clinical examinations

**DOI:** 10.1186/s41077-021-00179-9

**Published:** 2021-08-21

**Authors:** Mairead Corrigan, Helen J. Reid, Pascal P. McKeown

**Affiliations:** 1 Centre for Medical Education, Queen’s University Belfast, Whitla Medical Building, 97 Lisburn Road, Belfast, BT9 7BL UK; 2 School of Medicine, Dentistry and Biomedical Sciences, Queen’s University Belfast, Whitla Medical Building, 97 Lisburn Road, Belfast, BT9 7BL UK

**Keywords:** Simulated/standardised participants, Education, Objective structured clinical examination

## Abstract

**Background:**

Simulated participants (SPs) play an important role in simulated assessments of clinical encounters between medical students and patients, most notably in objective structured clinical examinations (OSCEs). SP contributions to OSCEs are invaluable, taking the role of a patient or carer. While SPs in some settings/contexts may rate students, their role has been problematized in the literature for their lack of agency within a standardised format of OSCEs that promotes reliability, objectivity and accountability. In this study, we explored SP experiences for tensions that result from simulated assessments and their potential implications for education.

**Methods:**

Semi-structured interviews were conducted with seven SPs who were also tasked with providing a global mark for students. They were purposively selected to include women and men of different ages, occupation, education and experience as an SP. The interviews were analysed using a critical thematic analysis using a phenomenological approach.

**Results:**

SP experiences directly addressed tensions and contradictions around OSCEs. SP participants described their experiences under four themes: industrialising, reducing, performativity and patient safety. OSCEs were compared to an industrial process that promoted efficiency but which bore no resemblance to real-life doctor-patient encounters. They were perceived to have a power and agency that reduced SPs to verbalising scripts to ensure that students were exposed to a standardised simulated experience that also underlined the performative role of SPs as props. These performative and reductionist experiences extended to students, for whom the mark sheet acted as a checklist, promoting standardised responses that lacked genuineness. All of this created a tension for SPs in promoting patient safety by ensuring that those medical students who passed were clinically competent.

**Conclusions:**

OSCEs often form part of high-stakes exams. As such, they are governed by processes of industrialisation, accountability and standardisation. OSCEs possess a power and agency that can have unintended negative consequences. These include ‘conditioning’ students to adopt behaviours that are not suited to real-life clinical encounters and are not person-centred.

## Background

‘Why didn’t they see my scars?’

This simulated participant (SP) had had both a Caesarean section and an open cholecystectomy, with two obvious and large (> 10 cm) abdominal scars. She questioned the examiner as to why a succession of medical students, undertaking an objective structured clinical examination (OSCE), had ignored her scars. The students had variously stated, ‘there are no scars, spider naevi or other signs of chronic liver disease.’ This contradiction led the authors to explore SP lived experiences of OSCEs, as a relatively under-voiced group, using critical thematic analysis [1].

Since their introduction in 1979 in medical education [2, 3], OSCEs have become a main method of assessing clinical skills in simulated environments across health professions education (HPE). In OSCEs, examiners score students’ clinical competence across a variety of fixed-interval ‘stations’ (usually 5 or 10 min each) that simulate clinical scenarios, some of which include SPs. The role of SPs has expanded from playing patients to portraying carers and health professionals in a standardised way and may include rating students on various assessment instruments [4]. The popularity of OSCEs is derived from discourses of performance, psychometrics and production in delivering an assessment that is characterised respectively by fairness, reliability and validity and accountability [5–8].

More recently, studies have begun to question the educational impact of highly standardised OSCEs for student learning. The use of checklists for scoring students have been criticised for elevating competency over empathy and for undermining person-centred care [9–11]. The expansion of the SP role in simulated assessments and learning environments has been accompanied by a critique of their lack of power and agency and their objectification as props [10, 12, 13].

In this study, a critical thematic analysis using a phenomenological approach was used to explore SP lived experiences of the phenomenon that is OSCEs. We explored OSCE tensions and contradictions, like the one experienced by the SP in the opening paragraph.

We were interested in SPs’ experiences to expose hidden tensions in the simulated assessment environment of OSCEs, with a view to generating changes in HPE assessment practices to benefit learners and, ultimately, patients.

## Methods

This study was approved by the School of Medicine, Dentistry and Biomedical Sciences Research Ethics Committee in Queen’s University, Belfast (ref 15.39).

### Setting

The setting was the undergraduate medical degree programme at Queen’s University, Belfast, which has an annual intake of 262 students. SPs play the role of patients and allocate a mark for students in simulated learning environments and assessments across all 5 years of the curriculum.

### Research team and reflexivity

HR and PMcK are practising clinicians and OSCE examiners. MC is a sociologist working within HPE, and an examiner for multiple mini interviews, otherwise known as ‘admissions OSCEs’ [14]. All team members are experienced qualitative researchers. HR was responsible for recruiting and interviewing the SPs. Her prior contacts with some of the SPs as an OSCE examiner led her to focus recruitment at SPs who she had not worked with. She reflected on the potential power dynamic of her position as an OSCE examiner after each SP interview. All the researchers considered their subjectivities and positionalities during data analysis, in keeping with a phenomenological approach.

### Recruitment and sampling

**Fig. 1:**
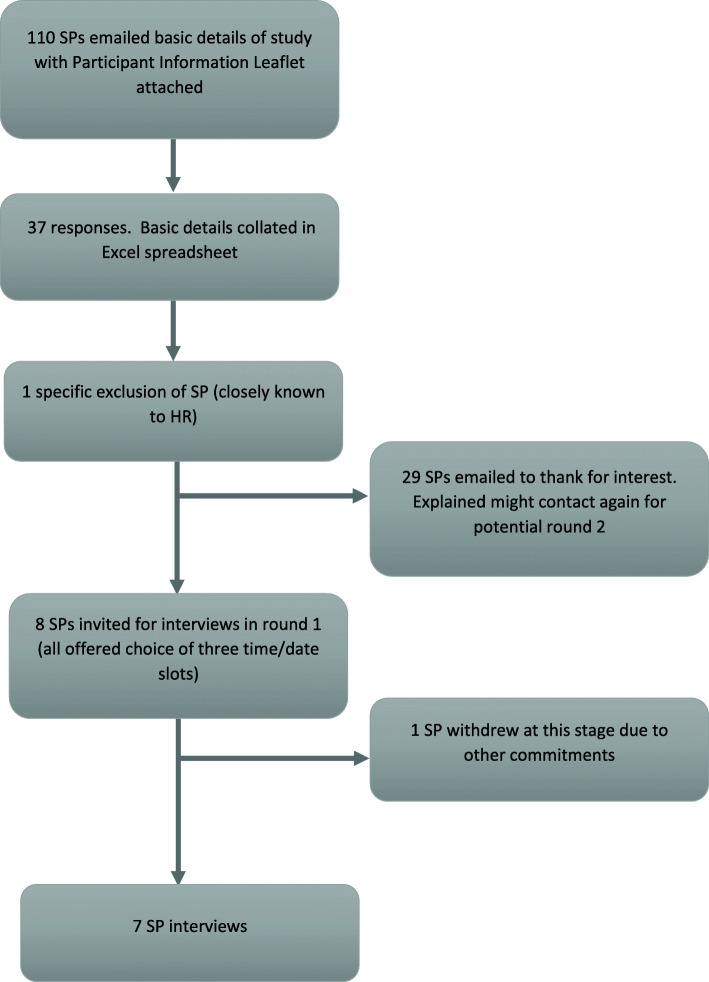
Recruitment flowchart

All individuals registered with Queen’s University Belfast (QUB) as SPs (*n* = 110 at time of study) were invited by e-mail to participate. HR purposively selected a maximum variation sample from the 37 individuals who responded affirmatively, to include different genders, ages, level of experience as an SP and prior professional experience (Fig. 1). She interviewed five female and two male SPs, aged between 40 and over 70. Their experience as SPs ranged between less than 2 years (1 SP) to more than 10 years (3 participants). Participants gave voluntary and informed written consent and confidentiality was maintained.

### Data collection

**Fig. 2:**
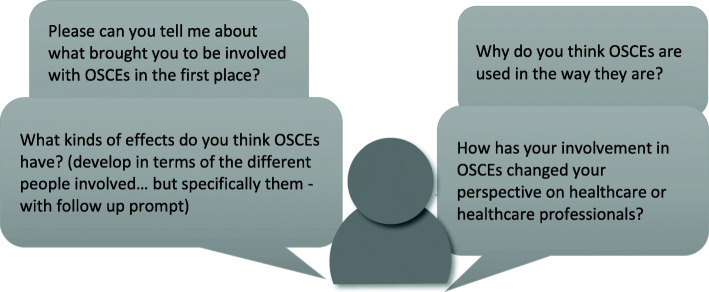
Interview schedule

Data was collected with seven participants with flexibility to schedule further data collection. This was not deemed necessary as the team felt that no new issues had emerged in the latter interviews. The semi-structured interview questions (Fig. 2) were constructed iteratively.

The interview schedule was used to guide discussions, with HR following up on participant responses with prompts and probing and with non-verbal cues such as nodding to encourage further unprompted input. Participants were encouraged to discuss the challenges and unintended consequences of OSCEs and their future use. Space was provided for ‘the doorknob comment’ and participants were encouraged to discuss information relevant to the research that was not covered by the interview schedule. Interviews were audio-recorded on two devices and they ranged from 25 to 80 min in duration. HR transcribed the data. As interviewer, she remained aware of the identity of all study participants but ensured the transcripts were anonymised prior to circulation among the wider research team (see ‘Availability of data and material’).

### Data analysis

Our thematic analysis was inductive and iterative, occurring simultaneously with data collection. Analysis involved reading the transcripts several times to promote familiarity, followed by coding and memo-writing, aided by the data software package NVivo. All members of the research team participated in the analysis.

## Results

SPs’ experiences addressed tensions and contradictions around OSCEs under four themes: industrialising, reducing, performativity and patient safety.

### Industrialising

SPs used factory metaphors to compare OSCEs to an industrial process that bears no resemblance to real-life doctor-patient encounters. They spoke with a sense of awe about the scale of OSCEs in terms of the number of students and the logistics of ensuring that every stage of the process operates like a well-oiled machine. This analogy between OSCEs and machines was achieved by SPs’ use of the verb ‘to run’ to describe the whole process, invoking the forward motion of cogs in a machine. They elevated the role of machines, in this case computers, for producing the final results from examiners’ scores and scribbled notes of students’ performance as if the computer itself had some agency. Other parallels were the economic advantages of OSCEs as the most cost-effective and efficient way to assess a large number of students over a relatively short space of time. The close monitoring of students to ensure that everything goes smoothly led SPs to emphasise the dual role of OSCEs as a method of assessment and surveillance.

I think the OSCEs here are incredibly well run, very well run, a complete logistical nightmare, I’ve no idea how they do it…. I know it all goes through the computer and all the scores and all that and then they go in a pile and then they go somewhere… Actually an OSCE is probably the most cost-effective way of being able to try and monitor and assess 260 people. (SP6)

The amount of preparation and planning, and the precision with which OSCEs are carried out, encouraged SPs to liken them to military operations. One SP compared how medical OSCEs were more ‘disciplined’ than police recruitment procedures. The factory metaphors continued with the SP describing how medical OSCEs are driven ‘by the clock’ and ‘like clockwork’, similar to how factory workers ‘clock on and off’. Their description of how a large number of students passed through the OSCE station asking the same questions and giving the same diagnosis so that ‘by the third or fourth one I know what I have’ has similarities to the monotony of a factory assembly line. The use of the verb ‘to do’ twenty of them in a similar vein serves to describe their interactions with the students as mechanistic.

I mean the way they run the OSCEs here, is very, I’ve worked for the police as well you see, and the police one I was expected to be regimented and very good and disciplined and everything, yet I find that here, in the medical err-place, more, eh disciplined and really by the clock and everything is like clockwork and very military as it were…I mean the point about it is, although I don’t know what the doctors have to say, but if you do twenty of these guys, you get a gist of…What they’re supposed to be saying…. I mean, if I’ve gout, by the third or fourth one I know what I have! (SP2)

### Reducing

SPs used a reductionist metaphor to describe their role in OSCEs that was limited to verbalising scripts promoting uniformity. Improvisation was prohibited in favour of patients being portrayed in the same standard way to enhance students’ chances of passing. This was regarded as more favourable to students compared with how students used to be assessed with ‘real’ patients who could be unpredictable in their behaviour, thereby increasing students’ chances of failing. In the extract below, the SP’s empathy with the students when they used the second person ‘you’ and their concern that students be given an equal chance of passing encouraged them to accept their role being reduced to learning scripts.

it’s to have kind of uniformity about it, that they all try to do the same thing, stick to the same script, that every student gets a bit of uniformity, rather than, people going round real wards where you might be desperately unlucky with the patient you land, and your prospects damaged by it, you know with an examiner going round with you, you know, perhaps it was like that in the old days. (SP5)

OSCE scripts were tightly bounded by rules, imparted to them in their training that did not allow SPs to reveal too much to students that would give them an advantage. SPs could only provide certain information ‘if it’s within the rules’ (SP7).

There’s certain things you just cannot not say, if you know what I mean…That’s what they tell you. Not to give them that information, not to do this… (SP2)

The reductionist nature of OSCE scripts extended to students. One SP drew parallels between learner drivers and medical students, with both having to replace their natural behaviours with formal, contrived behaviours in order to pass their respective driving and ‘doctoring’ tests. While SPs were critical of how OSCEs had a power and agency that forced students to take on a persona, they accepted this as necessary for them to progress.

the thing is, it’s like learning to drive a car, you’re saying ‘ten to two’ with your hands… I mean who the hell drives ‘ten to two?! So if you wanna pass your OSCEs you have to say all those things. You may never use half of those things the medical things, you just say ‘ah thank you, so terrible, it’ll be alright’ but you still have to say it. So the environment… although very stilted and very strict and whatever, it’s for a reason. It’s like the doctoring test. (SP2)

SPs were empathetic to the ‘difficult’ situation of the students, who resigned themselves to playing tightly scripted roles as though they had been ‘conditioned’ to do so.

it is very difficult, but by and large they all seem to be very conditioned by it, they know exactly what to do. (SP6).

### Performativity

A powerful influence in conditioning students’ behaviour was the mark sheet that examiners use to assess students’ performance. OSCEs were described as a tick box exercise, where certain behaviours (regardless of whether or not they were experienced as meaningful or genuine by the SP) are ‘ticked off’ on a checklist, disaggregating students’ consultation skills into discrete boxes of which there may be a lot more for clinical skills than there are for psychological and communication skills. Marking students against a checklist disadvantaged students who immersed themselves in the scenario and displayed genuine empathy. In the extract below, the SP was critical of the mark sheet for promoting care that was not person-centred. They recounted their concern for a student who spent a lot of time providing emotional support for their character who had just received a diagnosis of a condition that was likely to prove fatal in a very short time and who was at the hospital clinic alone, because the SP knew that they were ‘not ticking off the examiner’s boxes’. They voiced concerns when students, displaying checklist driven OSCE behaviours, received more marks for their gamesmanship than students who displayed ‘genuine’ behaviours. Referring to students who ‘can bluff’ emphasised the performative nature of OSCEs.

in fact there was one guy that was so upset for me I thought he was going to offer to drive me home. And all I was thinking was ‘he’s not ticking off the examiner’s boxes. The sad thing is, if you have somebody who’s not knowledgeable but can bluff and knows what tick boxes to tick, then they can probably do alright. (SP6)

Students who were ‘playing to an OSCE script’ ran the risk of responding inappropriately to news of the death of a relative of the character played by an SP:

you say your father died when he was in his 50s of heart disease and people going, ‘good, good, good, good’ I’m seeing this happen! (SP5)

And he said to me, right, in one of the history taking he had to ask about my mum or something and my mum was supposed to be dead, and then I said ‘oh yeah my mum died a couple of days back’ and he said ‘fantastic!’ (SP2)

Not all SPs were critical of students putting on a performance. Some admitted that they would encourage students to do so. The advice to students by the SP below depicts OSCEs as a simulacra in which students can ‘bluff’ the examiner about their clinical competence by appearing confident, even though there was no proof of this. In providing this advice, the SP portrayed themselves as an ally to students that has the potential to undermine their objectivity.

And if I had to give any student advice beforehand it would be, to sound confident. Even if you don’t know what you’re talking about, sound as if you do. (SP5)

SPs’ observations of students practising what they were going to say before entering an OSCE station, paralleled performers rehearsing lines before going on stage.

I can see somebody waiting at the cubicle opposite and I’ve seen them almost, sort of, you know, you can see their lips moving, they’re kind of ((sharp inhalation)) going over their bits of knowledge. (SP4)

OSCEs as performative also came through in how SPs voiced their role as ‘I’m only there as a prop’ (SP3; SP6). The word ‘prop’ reinforced how they perceived their role as ancillary to the students as the main actors. It also served to objectify them as inanimate and agency-less objects. Use of the word ‘only’ emphasised the SP role as one-dimensional and minor.

### Patient safety

SPs perceived themselves as part of the health professions education team in promoting patient safety by ‘protecting the public’ from students who did not demonstrate clinical competence. The ‘we’ denoted an equality of roles between SPs and examiners, who collectively ensured that only students, who were competent, pass. Their agency in promoting patient safety stemmed from their allocation of marks for how well students interacted with them as a patient. It also reflected their enhanced medical knowledge from their experience of acting the role of the patient. They acknowledged though the tension between their public duty to promote patient safety and their role in supporting students to pass.

we would say the most important thing is that we sift out the doctors that just aren’t cutting it, from the ones that are gonna cut it, you know? Umm, and so there is that thing you know. Whereas, I’ve spouted about all this ‘it’s all about the student and all’ I suppose there’s an extent to which…… it’s about protecting the public as well because you are trying to sift out the ones who are rubbish. (SP3)

Being an advocate for both students and patients was a balancing act for SPs. They were indignant when students ignored physical scars that were real because this was symbolic of their objectification as props. However, they put this within the context of OSCEs as simulated clinical scenarios that led them to turn a blind eye if the rest of the students’ physical examination was a textbook demonstration that reassured them of the students’ clinical competence.

I have a caesarean scar and it’s always missed! But the important thing there from where I’m standing, or sitting, not only an SP but as … a patient, is, as long as they know in their head exactly how they’re supposed to divide up the abdomen, and how they’re palpating it, and the manner in which they’re doing so and what they’re telling you to do whether roll onto your right, or roll onto your left, at least I know that clinically they know what they’re doing! (SP6)

## Discussion

Unlike studies that have problematised the industrialisation of OSCEs [10, 15], SPs lauded the precision and efficacy of OSCEs in assessing large numbers of students by using military and industrial analogies. They viewed the mechanistic nature of OSCEs as advantageous for promoting efficiency, fairness and cost-effectiveness. They were content for their role-playing to be standardised and controlled by rules and tightly bounded scripts in promoting fairness to all students. Their discussions of standardisation extended to the students, when they accepted the power and agency of OSCEs to ‘monitor’ and ‘condition’ students’ behaviour that has echoes of Foucault’s panoptic surveillance [16]. They saw merit in having a checklist of core clinical skills for promoting patient safety which they perceived themselves as playing a key role as members of the health professions education team. They accepted how, like learning to drive a car, learning to be a doctor involved students performing unnatural behaviours in training and exams which they invariably adapt in real-life situations. Training learners in one set of discourses and examining them in another does little to prepare learners for real-world practice [11]. Yet a critical analysis of SP experiences revealed some tensions, contradictions and unintended behaviours of OSCEs. Mark sheets were perceived to advantage students who performed to the checklist and disadvantage students who displayed genuine behaviours. They gave rise to unintended consequences with students not listening intently and responding inappropriately to SP revelations of bereavement. The performative nature of OSCEs encouraged a rigid, rehearsed set of communication behaviours and favoured students who could ‘bluff’ their clinical knowledge and pass. Similar unintended consequences have been identified with the safety checklist used in surgery [17] with theatre teams adopting ‘work-around’ strategies [18, 19] or feeling a ‘false sense of security’ [20]. Another tension that emerged was the treatment of SPs as props that was objectifying and performative. SPs were keen to promote their agency through their sense of responsibility for maintaining patient safety and the allocation of a global mark to students based on how well they interacted. This had the potential to produce a conflict of interest with SPs wanting students to pass and a public responsibility that they felt to maintain patient safety by not being too generous with their marks for students who are not clinically competent. This expands on previous studies which were critical of the objectification of SPs similar to Baudrillard’s ‘simulacra’, a postmodern concept that describes how a copy that is indiscernible from the original replaces it by becoming the new reality — in this case, SPs replacing the manikins that students traditionally use to practise clinical skills [13].

### Future implications for undergraduate medical training

Further standardisation of OSCEs is a real possibility with the introduction of the Medical Licensing Assessment (MLA), which all medical graduates in the UK in 2024/2025 must pass. This could be due to increased standardisation. The potential for medical schools to be ranked by performance in standardised assessments could encourage ‘teaching to the test’ and a preoccupation with training students to perform well in OSCEs. The ‘rotes and routines’ that this would engender, the potential dissociation of looking and seeing, hearing and listening, hold with them the possibility of damaging future patient care. Medical students would become attuned to normality in rehearsing such routines on each other and in simulated and controlled environments. Exposure to the mess and uncertainties of authentic clinical practice would be minimised as it does little to prepare students for OSCEs.

### Strengths and Limitations

This research was contextually situated within a single UK institution, and we do not claim to present generalisable findings. By providing a comprehensive account of the context and conceptual approach, however, we believe that our findings are relevant and transferrable to others working across health professions education. Although we aspired to maximum variation in sampling, we recognise that the QUB SPs display demographic homogeneity, with the majority being women, retired, white and from professional backgrounds. In 2005, Frank posed an important question for qualitative researchers: ‘What can one person say about another? Research is, in the simplest terms, one person’s representation of another’ [21]. Qualitative research is open-ended: other voices will interact with it and develop the findings. Our findings are, inevitably, constructions of SP experiences through our various perspectives as doctors, researchers and examiners, which we invite readers to consider and build upon.

## Conclusions

OSCEs are often high-stakes exams. As such, they are governed by processes of industrialisation, accountability and standardisation. OSCEs possess a power and agency that can have unintended negative consequences. These include ‘conditioning’ students to adopt behaviours that are not suited to real-life clinical encounters and are not person-centred. OSCEs are a performance that can encourage students to ‘bluff’ with the potential consequential risks to patient safety. These unintended consequences of OSCEs reinforce the need for policy-makers and regulators to ‘take account of the educational effects of their assessments’, recognising the catalytic potential of assessment on education and healthcare systems [22].

## Data Availability

An example transcript (redacted for individually identifiable features) from the original dataset supporting the conclusions of this article is available in the Zenodo repository, 10.5281/zenodo.4311454. Further data may be obtained by contacting the corresponding author.

## Abbreviations

HPE: Health professions education
OSCE: Objective structured clinical examination
QUB: Queen’s University Belfast
SMDBS: School of Medicine, Dentistry and Biomedical Science
SP: Simulated participant

## References

[1] Braun V, Clarke V (2006). Using thematic analysis in psychology. Qualitative Research in Psychology..

[2] Harden RM, Stevenson M, Downie WW, Wilson GM (1975). Assessment of clinical competence using objective structured examination. Br Med J..

[3] Harden RM, Gleeson FA (1979). Assessment of clinical competence using an objective structured clinical examination (OSCE). Med Educ..

[4] Lewis KL, Bohnert CA, Gammon WL, Hölzer H, Lyman L, Smith C (2017). The Association of Standardized Patient Educators (ASPE) standards of best practice (SOBP). Adv Simul (Lond).

[5] Hodges BD (2009). The objective structured clinical examination a socio-history (second edition).

[6] Brailovsky CA, Grand'Maison P (2000). Using evidence to improve evaluation: a comprehensive psychometric assessment of a SP-based OSCE licensing examination. Adv Health Sci Educ Theory Pract..

[7] Van Der Vleuten CP (1996). The assessment of professional competence: developments, research and practical implications. Adv Health Sci Educ Theory Pract..

[8] Schuwirth LW, van der Vleuten CP (2011). General overview of the theories used in assessment: AMEE guide no. 57. Med Teach..

[9] Gormley GJ, Johnston JL, Cullen KM, Corrigan M. Scenes, symbols and social roles: raising the curtain on OSCE performances. Perspect Med Educ. 2021;10(1):14–22. 10.1007/s40037-020-00593-1. 10.1007/s40037-020-00593-1 PMC7809074 32504445

[10] Gormley GJ, Hodges BD, McNaughton N, Johnston JL (2016). The show must go on? Patients, props and pedagogy in the theatre of the OSCE. Med Educ..

[11] Hodges B (2003). OSCE! Variations on a theme by Harden. Med Educ..

[12] Kearney GP, Gormley GJ, Wilson D, Johnston JL (2018). Blurred boundaries: sexuality and power in standardised patients' negotiations of the physical examination. Adv Simul (Lond).

[13] Johnston JL, Kearney GP, Gormley GJ, Reid H. Into the uncanny valley: simulation versus simulacrum? Med Educ. 2020;54:903–7. 10.1111/medu.14184. 10.1111/medu.14184 32314435

[14] Eva KW, Rosenfeld J, Reiter HI, Norman GR (2004). An admissions OSCE: the multiple mini-interview. Med Educ..

[15] Johnston JL, Lundy G, McCullough M, Gormley GJ (2013). The view from over there: reframing the OSCE through the experience of standardised patient raters. Med Educ..

[16] Foucault M (1977). Discipline and punish : the birth of the prision.

[17] Haynes AB, Weiser TG, Berry WR, Lipsitz SR, Breizat AH, Dellinger EP (2009). A surgical safety checklist to reduce morbidity and mortality in a global population. N Engl J Med..

[18] Kitto S (2010). Evidence-based checklists: intended and unintended consequences for interprofessional care. J Interprof Care..

[19] Urbach DR, Govindarajan A, Saskin R, Wilton AS, Baxter NN (2014). Introduction of surgical safety checklists in Ontario. Canada. N Engl J Med..

[20] Rydenfält C, Ek Å, Larsson PA (2014). Republished: Safety checklist compliance and a false sense of safety: new directions for research. Postgrad Med J..

[21] Frank AW (2005). What is dialogical research, and why should we do it?. Qual Health Res..

[22] Norcini J, Anderson B, Bollela V, Burch V, Costa MJ, Duvivier R, Galbraith R, Hays R, Kent A, Perrott V, Roberts T (2011). Criteria for good assessment: consensus statement and recommendations from the Ottawa 2010 Conference. Med Teach..

